# Obesity Bias in the School Setting: A Brief Report

**DOI:** 10.3390/children9071067

**Published:** 2022-07-18

**Authors:** José I. Baile, María J. González-Calderón, María F. Rabito-Alcón

**Affiliations:** Department of Psychology, Open University of Madrid (UDIMA), 28400 Madrid, Spain; mariajose.gonzalez@udima.es (M.J.G.-C.); mariafrenzi.rabito@udima.es (M.F.R.-A.)

**Keywords:** obesity, obesity bias, children, discrimination, school setting

## Abstract

Obesity bias is one of the main psychosocial consequences experienced by people who are overweight and people with obesity. Therefore, its study, especially during childhood, has become an emerging objective. The aim of this study is to examine obesity bias in children in the school setting. In total, 171 primary school students (Mean age: 10.68; SD: 0.63) from a school in Madrid (Spain) filled out a survey in which they indicated whether they would choose a classmate with obesity with whom they would carry out academic, social, and leisure activities. The rejection ratios of peers with obesity and other personal characteristics such as gender, nationality, or ethnicity were compared. The results indicate that more than half of the participants would not choose a partner with obesity to carry out any of the three activities suggested, and that obesity was the personal characteristic that elicited the highest rate of rejection, especially among females. The possible explanations for these findings are discussed, as well as why the school setting should be a nonaggressive but protective environment for children with obesity.

## 1. Introduction

Obesity is one of the main health problems worldwide, which has physical, psychological, and social consequences within the lifespan [1,2]. In recent years, one of the main psychosocial aspects studied in obesity is the possible discrimination manifested by negative thoughts, behaviors, and attitudes towards people with obesity. In this sense, research has shown that there are stigmatizing attitudes towards people with obesity in the general population; for example, 23.5% of the subjects who participated in a study in Germany were found to have those stigmatizing attitudes [3]. Moreover, stigmatization and discrimination due to obesity is an international phenomenon, as confirmed by a transnational study of 2866 subjects from Canada, the USA, Iceland, and Australia, in which prejudice against people with obesity was found to be present in all these populations [4]. 

Stigmatization related to excess weight may involve an additional risk to the individual’s health, both physically and psychologically, and seems to reduce the probability that people discriminated against will request proper healthcare [5]. The emerging literature on this topic has highlighted the following consequences associated with obesity bias: interpersonal difficulties (relating to peers, establishing friendships, starting a romantic relationship, etc.) [6]; victimization (being insulted, attacked, or mobbed) [7], one in three children who are bullied seem to be overweight [8]; psychological issues (low self-esteem, body dissatisfaction, and a high risk of further psychopathology) that last beyond childhood, common in adults who were obese as children [9]; poor health-related quality of life [10]; and consequences in the academic and occupational domains (poor academic results not due to a lack of cognitive skills) [11]. The belief that children with obesity are less competent academically is a self-fulfilling prophecy due to the fact that the way teachers and classmates behave, in terms of expectations, collaboration, and involvement, favors poor academic performance in children with obesity in the long run.

Obesity bias has been found in different settings, especially in educational [12], medical [13], and work [14], as well as within the families of stigmatized individuals [15]. The school setting is the place where children and adolescents with obesity seem to suffer the worst effects of stigmatization, and it is precisely the environment in which they spend a large amount of time and establish multiple interactions that reveal prejudices related to physical appearance, such as academic or leisure group activities, sports, or extracurricular activities, in which children and adolescents share spaces (changing room, cafeteria, toilet, etc.). In fact, it is in the school setting where males with obesity remember their first rejections regarding their appearance occurring, whereas females report their first rejections usually taking place in the family setting [16].

Prejudice and stigmatization appear from an early age. Evidence of bias towards overweight peers has been found as early as three years of age [17]. In this sense, there is a study that analyzed the prejudice towards peers with obesity among children aged 10 and 11, in which children with obesity were found to be the ones who elicited the least sympathy compared to peers with certain kinds of physical disabilities [18]. This research carried out in 1961 was repeated 40 years later. The new results were not only replicated, indicating little sympathy for children with obesity, but also showed that the distance of the evaluations on children with obesity with respect to the other groups had increased by 41% [19], which may indicate that prejudices and stigmatization regarding obesity have been increasing in the last decades.

Considering the information provided and the fact that expanding knowledge about obesity bias and the way to reduce it are considered universal health-related challenges [20], the present study aims to examine obesity bias in a sample of Spanish primary school children, which is a country and developmental stage in which it has rarely been studied.

## 2. Materials and Methods

### 2.1. Sample

A convenience sample of 171 Spanish students from a primary school in Madrid (Spain) (mean age: 10.68; SD: 0.63) took part in the study, 54.98% of whom were males. They were asked to participate anonymously on a voluntary basis and not to provide any identification information. The inclusion criteria were: (1) to complete the form in full and (2) to provide their parents’ informed consent to participate in the study.

### 2.2. Survey Administration

The study was carried out following the ethics criteria of the Open University of Madrid. It was approved on 24 April 2016 by the committee responsible for Research Ethics (registration number 1042016). Similarly, informed consent was obtained from all participants in writing and signed by their parents or legal guardians. 

Participants were administered a questionnaire that evaluated different aspects related to obesity, among these were several items about their choice of peers to carry out different activities together, academic (school homework), social (excursion), and leisure (playing videogames). These three activities were chosen because they are representative of the spheres of social interaction in which children spend the most time at school or outside school with their classmates (playing, doing academic tasks, etc.) Other types of interactions, such as family interactions, were not assessed, because the study aimed to analyze children’s discrimination only in the school setting.

The participants had to indicate whether or not they would choose each of the following type of schoolmate for each of the three proposed activities: a student from another country who speaks the same language (nationality); a schoolmate with obesity (obesity); a student from a different ethnic group or skin color (ethnic group); a very thin schoolmate (thinness), or a student of another sex (sex).

It was expressly stated that participation was voluntary, and that the information collected would be confidential and used for research purposes only, in accordance with Spanish Law on the Protection of Personal Data. Responses were coded anonymously using individual identification numbers. Anonymity, together with the fact that no participants received any academic benefit for their participation, aimed to address potential sources of bias. 

### 2.3. Data Analysis

Since this study is part of a broader research project on psychosocial characteristics of childhood obesity, it is merely exploratory in nature and aims to confirm trends in discriminatory behavior towards children with obesity but not to draw statistical inferences from the results. Descriptive analyses were performed using a cross-sectional methodology. The percentage of subjects who did not choose peer with obesity to carry out the suggested activities together is used as an indicator of discrimination compared to the proportion of participants who did not choose students with other characteristics (different nationality, ethnic group, etc.). Data were coded and analyzed by means of the SPSS software package, version 21.0 for Windows.

## 3. Results

Table 1 shows the percentage of the total sample and that of males and females separately who did not choose a peer to carry out each of the suggested activities based on the characteristics under study. 

As can be seen, all the characteristics under study triggered some type of rejection or discrimination, with the minimum percentage of rejection or non-choice being 28.1%, corresponding to performing an academic task with a person with a different nationality. As far as the participants’ sex was concerned, obesity bias was more accentuated in females, who did not choose either males or females with obesity with whom to carry out any of the activities in a higher percentage than their male counterparts, especially leisure activities (61% vs. 50%).

**Figure 1:**
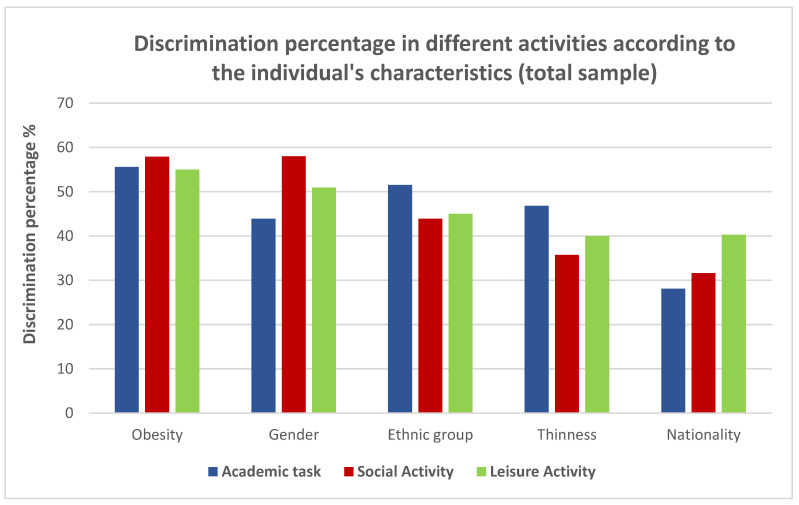
Percentage of participants from the total sample that did not choose a partner according to his/her characteristics for a specific activity (academic, social, or leisure).

Figure 1 reflects the level of discrimination (or non-choice) of the total sample in relation to each group analyzed. More than half of the sample did not choose a person with obesity for any of the activities, academic, leisure, or social. This obesity bias was higher than that reported for the peers’ nationality, ethnic group, or thinness. Only sex elicited a slightly higher rejection than obesity (58% versus 57.9%) to carry out social activities together.

## 4. Discussion

Preliminary data from this pilot study indicate there may be obesity bias within the school setting, since more than half of the participants did not choose a classmate with obesity for any type of activity, with the rejection rates higher than those found towards students with other characteristics (ethnic group, nationality, sex, etc.). Although this finding is in line with previous research that found the same trend of obesity discrimination in school environments, either among classmates or even by teachers [11,21,22], the results of the present study show the presence of obesity bias in a developmental stage and a country where this construct had rarely been studied before. However, these data cannot be interpreted as conclusive due to the sample size and the exploratory and descriptive nature of the statistical analysis.

The percentage of students who did not choose schoolmates of a different sex with whom to carry out leisure activities was slightly higher than that of children who did not choose a student with obesity for such activities, which could be explained by the age of the participants, since at this developmental stage it is common for males and females to choose members of the same sex to play and socialize with, a phenomenon known as sexual segregation [23]. It is also interesting to see how females at this early age are the ones who show greater rejection towards peers with obesity, which is consistent with the higher obesity stigmatization rates found in women in later developmental stages [24].

A surprising result is that all the groups under study elicited some type of rejection. This may be due to the research methodology used, since the subjects were required to state whether or not they would choose a schoolmate with a certain characteristic with whom to carry out one of the three activities suggested. The results may have been different if the participants had freely expressed the characteristics towards which they experienced rejection, but in that case, children may have not thought of obesity, which was the aim of the study.

Why does obesity elicit rejection even in childhood? Why do children reject their peers with obesity? The answer to these questions can be found in previous research, such as the study in which children were asked what they related childhood obesity to through qualifying adjectives; obesity at that early age was related to being lazy, careless, dirty, mischievous, cheating, lying, prone to quarreling, petty, ugly, and stupid [25,26]. Moreover, schoolchildren with obesity have been found to have greater difficulties in academic interactions, because their classmates tend to describe them as not suitable for certain social activities or lazy [27]. The overvaluation of thinness as an aesthetic reference, which has been proven to start from a very early age [28,29], may also play an important role.

It can be concluded that despite the limitations previously pointed out, the findings of this research suggest the existence of childhood obesity bias in school settings, as shown in previous research [30], as well as the need to carry out actions to identify and prevent it, as has already been proposed in the healthcare context [31], so that the negative psychosocial consequences of childhood obesity do not extend into adulthood [32]. It is important to confirm obesity bias in childhood in order to prevent its consequences as soon as possible, such as poor academic achievement, which probably explains why children with obesity are not chosen for academic tasks. In addition, it must be highlighted that discrimination based on obesity cannot be used as a strategy to reduce obesity itself, as other researchers have previously stated [33], since it may increase the risk of psychological issues, especially if it takes place in childhood, and it may worsen the child’s weight status, turning excessive eating into a kind of gratification to fight obesity bias.

A school setting should not be the place where discriminatory attitudes of any kind are manifested but a place that works to reduce discrimination, including that towards people with obesity. In that sense, we do believe that the proposals included in the recent international consensus statement for ending stigma of obesity [5] will help reduce discrimination in school contexts, such as “*treat overweight and people with obesity with dignity and respect; refrain from using stereotypical language, images, and narratives that unfairly and inaccurately portray overweight and people with obesity as lazy, gluttonous, and lacking willpower or self-discipline; encourage and support educational initiatives aimed at eradicating weight bias through the dissemination of current knowledge about obesity and body weight regulation; and encourage and support initiatives aimed at preventing weight discrimination in the workplace, education, and healthcare settings*”.

## Figures and Tables

**Table 1:** Percentage of the sample that did not choose a schoolmate according to his/her characteristics for a specific activity (academic, social, or leisure).

		Individuals’ Characteristics				
		Obesity	Nationality	Ethnic Group	Thinness	Sex
**Academic task**	Males	53.3 *	26.6	52.1	42.6	43.6
	Females	58.4 *	29.9	50.6	51.9	44.2
	Total sample	55.6 *	28.1	51.5	46.8	43.9
**Leisure activity**	Males	50	41.49	45.7	30	58.5 *
	Females	61 *	38.96	44.2	53	41.6
	Total sample	55 *	40.35	45	40	50.9
**Social activity**	Males	56.4 *	34	43.6	25.66	56
	Females	59.7	28.6	44.2	45.45	60 *
	Total sample	57.9	31.6	43.9	35.67	58 *

* Row maximum value.

## Data Availability

The data presented in this study are available on request from the corresponding author.
